# (2*E*)-2-[2-(Cyclo­hexyl­carbamothio­yl)hydrazinylidene]­propanoic acid

**DOI:** 10.1107/S1600536811014449

**Published:** 2011-04-22

**Authors:** Md. Abu Affan, Md. Abdus Salam, Fasihuddin B. Ahmad, Seik Weng Ng, Edward R. T. Tiekink

**Affiliations:** a Faculty of Resource Science and Technology, Universiti Malaysia Sarawak, 94300 Kota Samarahan, Sarawak, Malaysia; b Department of Chemistry, University of Malaya, 50603 Kuala Lumpur, Malaysia

## Abstract

In the title thio­urea derivative, C_10_H_17_N_3_O_2_S, the carboxyl group and the least-squares plane through the cyclo­hexyl ring are twisted out of the plane through the central CN_3_S residue; the respective dihedral angles are 7.18 (8) and 62.29 (4)°. The conformation about the azomethine bond [1.275 (2) Å] is *E*. The NH groups are *anti*, with one forming an intra­molecular N—H⋯N hydrogen bond. The main feature of the crystal structure is the formation of linear supra­molecular chains along [110] mediated by alternating pairs of O—H⋯O and pairs of N—H⋯S hydrogen bonds.

## Related literature

For related thio­urea structures, see: Normaya *et al.* (2011 ▶); Salam *et al.* (2011 ▶).
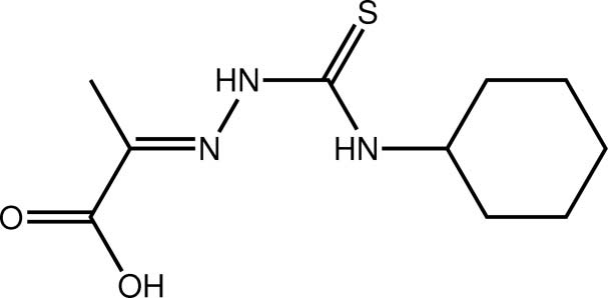


## Experimental

### 

#### Crystal data

C_10_H_17_N_3_O_2_S
*M* *_r_* = 243.33
Monoclinic, 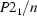

*a* = 8.9204 (2) Å
*b* = 6.0350 (2) Å
*c* = 22.4750 (6) Å
β = 90.051 (3)°
*V* = 1209.93 (6) Å^3^
*Z* = 4
Mo *K*α radiation
μ = 0.26 mm^−1^
*T* = 100 K
0.30 × 0.20 × 0.10 mm

#### Data collection

Agilent Supernova Dual diffractometer with an Atlas detector
Absorption correction: multi-scan (*CrysAlis PRO*; Agilent, 2010 ▶) *T*_min_ = 0.687, *T*_max_ = 1.000
7394 measured reflections
2723 independent reflections
2251 reflections with *I* > 2σ(*I*)
*R*_int_ = 0.036

#### Refinement

*R*[*F* ^2^ > 2σ(*F* ^2^)] = 0.041
*wR*(*F* ^2^) = 0.143
*S* = 0.98
2723 reflections
158 parameters
3 restraints
H atoms treated by a mixture of independent and constrained refinement
Δρ_max_ = 0.36 e Å^−3^
Δρ_min_ = −0.32 e Å^−3^

### 

Data collection: *CrysAlis PRO* (Agilent, 2010 ▶); cell refinement: *CrysAlis PRO*; data reduction: *CrysAlis PRO*; program(s) used to solve structure: *SHELXS97* (Sheldrick, 2008 ▶); program(s) used to refine structure: *SHELXL97* (Sheldrick, 2008 ▶); molecular graphics: *ORTEP-3* (Farrugia, 1997 ▶) and *DIAMOND* (Brandenburg, 2006 ▶); software used to prepare material for publication: *publCIF* (Westrip, 2010 ▶).

## Supplementary Material

Crystal structure: contains datablocks global, I. DOI: 10.1107/S1600536811014449/hb5851sup1.cif

Structure factors: contains datablocks I. DOI: 10.1107/S1600536811014449/hb5851Isup2.hkl

Additional supplementary materials:  crystallographic information; 3D view; checkCIF report

## Figures and Tables

**Table 1:** Hydrogen-bond geometry (Å, °)

*D*—H⋯*A*	*D*—H	H⋯*A*	*D*⋯*A*	*D*—H⋯*A*
N3—H3⋯N1	0.87 (1)	2.20 (2)	2.574 (2)	106 (2)
O1—H1⋯O2^i^	0.85 (1)	1.80 (1)	2.6416 (17)	172 (3)
N2—H2⋯S1^ii^	0.89 (1)	2.65 (1)	3.5384 (15)	176 (2)

Symmetry codes: (i) 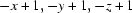; (ii) 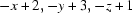.

**Fig. 1.**
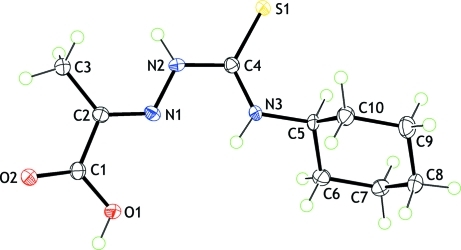
The molecular structure of (I) showing displacement ellipsoids at the 50% probability level.

**Fig. 2.**
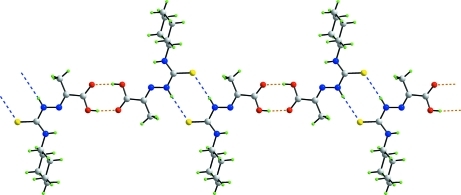
A view of the supramolecular chain aligned along [110] in (I). The O—H···O and N—H···S hydrogen bonds are shown as orange and blue dashed lines, respectively.

**Fig. 3.**
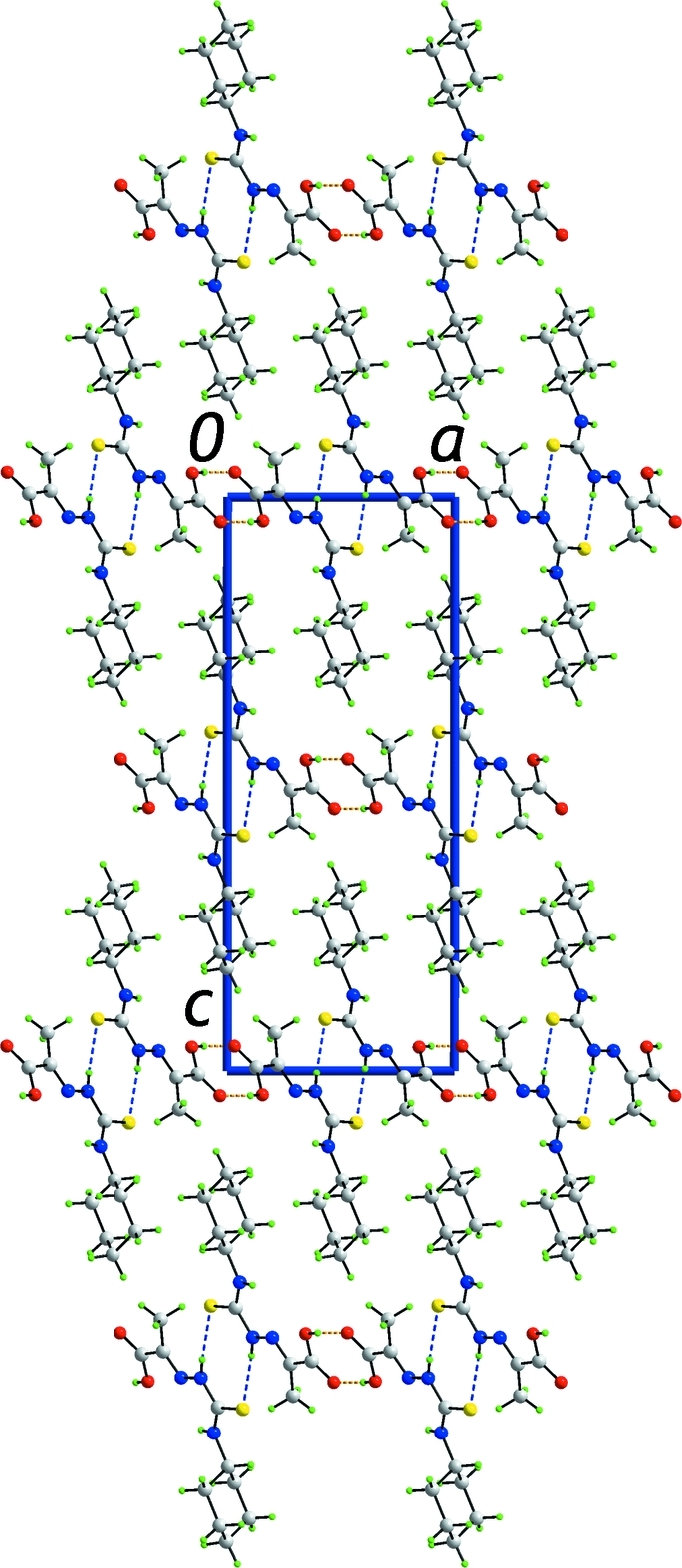
A view in projection down the b axis of the crystal packing in (I) showing the inter-digitation of the cyclohexyl rings along the c direction. The O—H···O and N—H···S hydrogen bonds are shown as orange and blue dashed lines, respectively.

## supplementary crystallographic information

### Comment

In continuation of structural investigations into conformation and hydrogen bonding in thiourea derivatives (Normaya *et al.* 2011; Salam *et al.*, 2011), the title compound, (I), was investigated.

The central CN_3_S chromophore in (I), Fig. 1, is planar (r.m.s. = 0.0039 Å). The carboxylate residue is slightly twisted out this plane (dihedral angle = 7.18 (8) °), and, by contrast, the least-squares plane through the cyclohexyl group (which has the conformation of a chair) is twisted significantly out of the central plane (dihedral angle = 62.29 (4) °). The H atoms of the NH groups are *anti*, and the conformation about the azomethine bond [1.275 (2) Å] is *E*. The N3—H forms an intramolecular hydrogen bond with the imino-N3 atom, Table 1. Finally, the thione and carboxylic acid groups lie to opposite sides of the molecule. This arrangements enables the formation of linear supramolecular chains *via* hydrogen bonds along [110] in the crystal packing, Fig. 2 and Table 1. The carboxylic acid residues self-associate *via* a centrosymmetric eight-membered {···HOC═O}_2_ synthon. Similarly, the thiourea entity with the NH atom not involved in the intramolecular N—H···N interaction, self-associates *via* a centrosymmetric eight-membered {···HNC═ S}_2_ synthon. Chains lie in the *ab* plane with the cyclohexyl rings inter-digitating along the *c* axis, Fig. 3.

### Experimental

Cyclohexylisothiocyanate (0.706 g, 5 mmol) and hydrazine hydrate (0.250 g, 5 mmol), each dissolved in 10 ml e thanol were mixed with constant stirring. The stirring was continued for 30 min and the white product that formed, *N*(4)-cyclohexylthiosemicarbazide, was washed with ethanol and dried *in vacuo*. A solution of this (0.51 g, 3 mmol) in 10 ml me thanol was refluxed with a methanolic solution of pyruvic acid (0.261 g, 3 mmol) for 5 h after the addition of 1–2 drops of glacial acetic acid. On cooling the solution to room temperature, white precipitate separated, which were filtered and washed with methanol. The white precipitate was recrystallized from methanol to yield colourless prisms and dried *in vacuo* over silica gel. (*M*.pt. 465–467 K. Yield 0.621 g (80%). Anal. Found: C, 49.31; H, 7.01; N, 17.18%. C_10_H_17_N_3_O_2_S requires: C, 49.36; H, 7.04; N, 17.26%. FT—IR (KBr, cm^-1^) ν_max_: 3322 (m, OH), 3197 (s, NH), 2922, 2851 (s, cyclohexyl), 1692 (m, C═O), 1619 (w, C═N), 980 (m, N—N), 1249, 873 (w, C═S).

### Refinement

Carbon-bound H-atoms were placed in calculated positions (C–H = 0.98 to 1.00 Å) and were included in the refinement in the riding model approximation, with *U*_iso_(H) set to 1.2–1.5*U*_eq_(C). The O– and N-bound H-atoms were located in a difference Fourier map and were refined with distance restraints of O—H = 0.84±0.01 Å and N—H 0.88±0.01 Å, and with *U*_iso_(H) = *yU*_eq_(N) for *y* = 1.5 (O) and 1.2 (N).

### Crystal data

**Table:**

C_10_H_17_N_3_O_2_S	*F*(000) = 520
*M**_r_* = 243.33	*D*_x_ = 1.336 Mg m^−^^3^
Monoclinic, *P*2_1_/*n*	Mo *K*α radiation, λ = 0.71073 Å
Hall symbol: -P 2yn	Cell parameters from 3312 reflections
*a* = 8.9204 (2) Å	θ = 2.3–29.1°
*b* = 6.0350 (2) Å	µ = 0.26 mm^−^^1^
*c* = 22.4750 (6) Å	*T* = 100 K
β = 90.051 (3)°	Prism, colourless
*V* = 1209.93 (6) Å^3^	0.30 × 0.20 × 0.10 mm
*Z* = 4	

### Data collection

**Table:**

Agilent Supernova Dual diffractometer with an Atlas detector	2723 independent reflections
Radiation source: SuperNova (Mo) X-ray Source	2251 reflections with *I* > 2σ(*I*)
Mirror	*R*_int_ = 0.036
Detector resolution: 10.4041 pixels mm^-1^	θ_max_ = 27.5°, θ_min_ = 2.5°
ω scans	*h* = −11→11
Absorption correction: multi-scan (*CrysAlis PRO*; Agilent, 2010)	*k* = −6→7
*T*_min_ = 0.687, *T*_max_ = 1.000	*l* = −29→29
7394 measured reflections	

### Refinement

**Table:**

Refinement on *F*^2^	Primary atom site location: structure-invariant direct methods
Least-squares matrix: full	Secondary atom site location: difference Fourier map
*R*[*F*^2^ > 2σ(*F*^2^)] = 0.041	Hydrogen site location: inferred from neighbouring sites
*wR*(*F*^2^) = 0.143	H atoms treated by a mixture of independent and constrained refinement
*S* = 0.98	*w* = 1/[σ^2^(*F*_o_^2^) + (0.1*P*)^2^] where *P* = (*F*_o_^2^ + 2*F*_c_^2^)/3
2723 reflections	(Δ/σ)_max_ = 0.001
158 parameters	Δρ_max_ = 0.36 e Å^−^^3^
3 restraints	Δρ_min_ = −0.32 e Å^−^^3^

### Special details

**Table:**

Geometry. All e.s.d.'s (except the e.s.d. in the dihedral angle between two l.s. planes) are estimated using the full covariance matrix. The cell e.s.d.'s are taken into account individually in the estimation of e.s.d.'s in distances, angles and torsion angles; correlations between e.s.d.'s in cell parameters are only used when they are defined by crystal symmetry. An approximate (isotropic) treatment of cell e.s.d.'s is used for estimating e.s.d.'s involving l.s. planes.
Refinement. Refinement of *F*^2^ against ALL reflections. The weighted *R*-factor *wR* and goodness of fit *S* are based on *F*^2^, conventional *R*-factors *R* are based on *F*, with *F* set to zero for negative *F*^2^. The threshold expression of *F*^2^ > σ(*F*^2^) is used only for calculating *R*-factors(gt) *etc*. and is not relevant to the choice of reflections for refinement. *R*-factors based on *F*^2^ are statistically about twice as large as those based on *F*, and *R*- factors based on ALL data will be even larger.

### Fractional atomic coordinates and isotropic or equivalent isotropic displacement parameters (Å^2^)

**Table:**

	*x*	*y*	*z*	*U*_iso_*/*U*_eq_	
S1	1.07001 (5)	1.46426 (7)	0.59008 (2)	0.01940 (18)	
O1	0.65476 (14)	0.6300 (2)	0.54143 (6)	0.0209 (3)	
O2	0.53180 (13)	0.7099 (2)	0.45694 (6)	0.0196 (3)	
N1	0.80028 (16)	1.0010 (2)	0.53480 (7)	0.0156 (3)	
N2	0.88614 (16)	1.1878 (2)	0.53543 (7)	0.0170 (3)	
N3	0.94326 (16)	1.0938 (3)	0.63114 (7)	0.0169 (3)	
C1	0.62814 (18)	0.7522 (3)	0.49413 (8)	0.0164 (4)	
C2	0.72145 (19)	0.9564 (3)	0.48899 (8)	0.0162 (4)	
C3	0.7098 (2)	1.0904 (3)	0.43363 (8)	0.0211 (4)	
H3A	0.6904	1.2455	0.4440	0.032*	
H3B	0.6273	1.0339	0.4091	0.032*	
H3C	0.8039	1.0800	0.4113	0.032*	
C4	0.96146 (18)	1.2376 (3)	0.58689 (8)	0.0156 (4)	
C5	1.00682 (18)	1.1176 (3)	0.69060 (7)	0.0157 (4)	
H5	1.1055	1.1951	0.6871	0.019*	
C6	1.0336 (2)	0.8900 (3)	0.71759 (9)	0.0210 (4)	
H6A	1.1037	0.8054	0.6921	0.025*	
H6B	0.9377	0.8076	0.7194	0.025*	
C7	1.0991 (2)	0.9113 (3)	0.78024 (8)	0.0221 (4)	
H7A	1.1116	0.7618	0.7977	0.026*	
H7B	1.1994	0.9811	0.7779	0.026*	
C8	0.9984 (2)	1.0501 (3)	0.82063 (9)	0.0235 (4)	
H8A	0.9019	0.9725	0.8267	0.028*	
H8B	1.0471	1.0689	0.8599	0.028*	
C9	0.9696 (2)	1.2771 (3)	0.79289 (9)	0.0250 (4)	
H9A	0.8988	1.3610	0.8182	0.030*	
H9B	1.0648	1.3611	0.7912	0.030*	
C10	0.9048 (2)	1.2561 (3)	0.73033 (8)	0.0212 (4)	
H10A	0.8046	1.1858	0.7325	0.025*	
H10B	0.8924	1.4055	0.7128	0.025*	
H1	0.602 (2)	0.514 (3)	0.5431 (12)	0.040 (7)*	
H2	0.892 (2)	1.275 (3)	0.5035 (6)	0.026 (6)*	
H3	0.888 (2)	0.976 (2)	0.6260 (11)	0.029 (6)*	

### Atomic displacement parameters (Å^2^)

**Table:**

	*U*^11^	*U*^22^	*U*^33^	*U*^12^	*U*^13^	*U*^23^
S1	0.0251 (3)	0.0177 (3)	0.0154 (3)	−0.00825 (16)	−0.0030 (2)	0.00088 (18)
O1	0.0233 (6)	0.0208 (7)	0.0185 (7)	−0.0089 (5)	−0.0048 (5)	0.0055 (6)
O2	0.0200 (6)	0.0205 (7)	0.0182 (7)	−0.0064 (5)	−0.0030 (5)	0.0018 (5)
N1	0.0148 (7)	0.0144 (7)	0.0178 (8)	−0.0030 (5)	0.0009 (6)	−0.0010 (6)
N2	0.0214 (7)	0.0140 (7)	0.0157 (8)	−0.0039 (6)	−0.0007 (6)	0.0036 (6)
N3	0.0196 (7)	0.0172 (7)	0.0140 (8)	−0.0068 (6)	−0.0047 (6)	0.0006 (6)
C1	0.0159 (8)	0.0178 (9)	0.0155 (9)	0.0010 (7)	0.0000 (7)	−0.0017 (7)
C2	0.0153 (8)	0.0168 (9)	0.0166 (9)	−0.0015 (6)	0.0005 (7)	0.0006 (7)
C3	0.0247 (9)	0.0215 (9)	0.0169 (9)	−0.0069 (7)	−0.0040 (7)	0.0022 (8)
C4	0.0154 (8)	0.0160 (8)	0.0155 (8)	−0.0002 (6)	0.0004 (7)	−0.0008 (7)
C5	0.0173 (8)	0.0184 (9)	0.0112 (8)	−0.0030 (7)	−0.0038 (6)	−0.0006 (7)
C6	0.0255 (9)	0.0168 (9)	0.0208 (9)	0.0006 (7)	0.0006 (8)	−0.0003 (8)
C7	0.0266 (9)	0.0186 (9)	0.0210 (10)	0.0007 (7)	−0.0032 (8)	0.0036 (8)
C8	0.0301 (10)	0.0263 (10)	0.0141 (9)	−0.0050 (8)	−0.0001 (8)	−0.0016 (8)
C9	0.0318 (10)	0.0241 (10)	0.0191 (9)	0.0032 (8)	−0.0004 (8)	−0.0057 (8)
C10	0.0255 (9)	0.0197 (9)	0.0185 (9)	0.0037 (7)	−0.0007 (8)	−0.0011 (8)

### Geometric parameters (Å, °)

**Table:**

S1—C4	1.6774 (18)	C5—C10	1.525 (2)
O1—C1	1.315 (2)	C5—H5	1.0000
O1—H1	0.845 (10)	C6—C7	1.530 (3)
O2—C1	1.225 (2)	C6—H6A	0.9900
N1—C2	1.275 (2)	C6—H6B	0.9900
N1—N2	1.363 (2)	C7—C8	1.528 (3)
N2—C4	1.370 (2)	C7—H7A	0.9900
N2—H2	0.892 (9)	C7—H7B	0.9900
N3—C4	1.330 (2)	C8—C9	1.527 (3)
N3—C5	1.458 (2)	C8—H8A	0.9900
N3—H3	0.870 (9)	C8—H8B	0.9900
C1—C2	1.491 (2)	C9—C10	1.525 (3)
C2—C3	1.488 (3)	C9—H9A	0.9900
C3—H3A	0.9800	C9—H9B	0.9900
C3—H3B	0.9800	C10—H10A	0.9900
C3—H3C	0.9800	C10—H10B	0.9900
C5—C6	1.520 (3)		
			
C1—O1—H1	113.7 (18)	C5—C6—H6A	109.5
C2—N1—N2	119.52 (15)	C7—C6—H6A	109.5
N1—N2—C4	117.71 (14)	C5—C6—H6B	109.5
N1—N2—H2	121.1 (14)	C7—C6—H6B	109.5
C4—N2—H2	121.2 (14)	H6A—C6—H6B	108.1
C4—N3—C5	124.97 (14)	C8—C7—C6	111.63 (15)
C4—N3—H3	120.2 (16)	C8—C7—H7A	109.3
C5—N3—H3	114.8 (16)	C6—C7—H7A	109.3
O2—C1—O1	124.08 (15)	C8—C7—H7B	109.3
O2—C1—C2	120.71 (15)	C6—C7—H7B	109.3
O1—C1—C2	115.18 (15)	H7A—C7—H7B	108.0
N1—C2—C3	126.77 (15)	C9—C8—C7	110.38 (16)
N1—C2—C1	114.80 (15)	C9—C8—H8A	109.6
C3—C2—C1	118.38 (15)	C7—C8—H8A	109.6
C2—C3—H3A	109.5	C9—C8—H8B	109.6
C2—C3—H3B	109.5	C7—C8—H8B	109.6
H3A—C3—H3B	109.5	H8A—C8—H8B	108.1
C2—C3—H3C	109.5	C10—C9—C8	111.43 (15)
H3A—C3—H3C	109.5	C10—C9—H9A	109.3
H3B—C3—H3C	109.5	C8—C9—H9A	109.3
N3—C4—N2	115.34 (15)	C10—C9—H9B	109.3
N3—C4—S1	124.83 (14)	C8—C9—H9B	109.3
N2—C4—S1	119.82 (13)	H9A—C9—H9B	108.0
N3—C5—C6	109.71 (14)	C5—C10—C9	111.06 (14)
N3—C5—C10	111.03 (14)	C5—C10—H10A	109.4
C6—C5—C10	110.79 (15)	C9—C10—H10A	109.4
N3—C5—H5	108.4	C5—C10—H10B	109.4
C6—C5—H5	108.4	C9—C10—H10B	109.4
C10—C5—H5	108.4	H10A—C10—H10B	108.0
C5—C6—C7	110.56 (15)		
			
C2—N1—N2—C4	175.80 (15)	C4—N3—C5—C6	−151.10 (16)
N2—N1—C2—C3	−0.7 (3)	C4—N3—C5—C10	86.1 (2)
N2—N1—C2—C1	−178.00 (14)	N3—C5—C6—C7	−179.54 (14)
O2—C1—C2—N1	169.21 (15)	C10—C5—C6—C7	−56.59 (19)
O1—C1—C2—N1	−8.9 (2)	C5—C6—C7—C8	56.5 (2)
O2—C1—C2—C3	−8.4 (2)	C6—C7—C8—C9	−55.6 (2)
O1—C1—C2—C3	173.52 (15)	C7—C8—C9—C10	55.2 (2)
C5—N3—C4—N2	−177.12 (15)	N3—C5—C10—C9	178.84 (14)
C5—N3—C4—S1	4.2 (2)	C6—C5—C10—C9	56.7 (2)
N1—N2—C4—N3	0.7 (2)	C8—C9—C10—C5	−56.1 (2)
N1—N2—C4—S1	179.49 (12)		

### Hydrogen-bond geometry (Å, °)

**Table:**

*D*—H···*A*	*D*—H	H···*A*	*D*···*A*	*D*—H···*A*
N3—H3···N1	0.87 (1)	2.20 (2)	2.574 (2)	106.(2)
O1—H1···O2^i^	0.85 (1)	1.80 (1)	2.6416 (17)	172 (3)
N2—H2···S1^ii^	0.89 (1)	2.65 (1)	3.5384 (15)	176.(2)

Symmetry codes: (i) −*x*+1, −*y*+1, −*z*+1; (ii) −*x*+2, −*y*+3, −*z*+1.
